# The relationship between the regional abdominal adipose tissue distribution and the serum uric acid levels in people with type 2 diabetes mellitus

**DOI:** 10.1186/1758-5996-4-3

**Published:** 2012-02-03

**Authors:** Tae Ho Kim, Seong Su Lee, Ji Han Yoo, Sung Rae Kim, Soon Jib Yoo, Ho Cheol Song, Yong-Soo Kim, Euy Jin Choi, Yong Kyun Kim

**Affiliations:** 1 Department of Internal Medicine, College of Medicine, The Catholic University of Korea, Seoul, Korea

**Keywords:** obesity, adiposity, diabetes mellitus, computed tomography, uric acid

## Abstract

**Background:**

Hyperuricemia is associated with obesity. The visceral adiposity and subcutaneous adiposity may be associated with the differential metabolic risk, and the distribution of abdominal adipose tissue was significantly altered in people with type 2 diabetes mellitus (DM) compared to healthy people. Our study was performed to determine to the association between the regional abdominal adipose tissue distribution and serum uric acid levels in people with type 2 DM.

**Methods:**

A total of 699 people with type 2 DM and who had undergone abdominal computed tomography assessment of the visceral fat area and subcutaneous fat area were included. The serum uric acid levels were measured by the uricase method. Hyperuricemia was defined by cut-off value of > 7 mg/dl for men and > 6 mg/dl for women.

**Results:**

The visceral fat area was positively associated with the serum uric acid levels after adjustment for age, sex, systolic blood pressure, diastolic blood pressure, serum creatinine, hemoglobin, serum albumin, serum high-density lipoprotein, serum triglyceride and hemoglobin A1c (β-coefficient = 0.117, *p *< 0.001). The logistic regression analysis showed that the visceral fat area was the significant independent predictor of hyperuricemia (OR 2.33, 95% CI, 1.21-4.50, p = 0.012). But there was no significant association between the subcutaneous fat area and the serum uric acid levels (β-coefficient = 0.061, *p *= 0.255).

**Conclusions:**

our data shows that the visceral fat area was positively associated with the serum uric acid levels, but the subcutaneous fat area was not in people with type 2 DM.

## Background

Hyperuricemia or elevated serum uric acid levels have been considered not only an independent risk factor for cardiovascular diseases but this also plays a role in the development of metabolic diseases [1-4]. Previous studies reported that the serum uric acid level is associated with the individual components of metabolic syndrome such as obesity, dyslipidemia and hypertension [5,6]. As for the association between the serum uric acid levels and obesity, a number of epidemiological and clinical studies have demonstrated a positive correlation between the serum uric acid levels and obesity [5,7].

The visceral fat component and the subcutaneous fat component may have differential metabolic risks [8]. The visceral fat component is metabolically active and it regulates numerous adipocytokines and other vasoactive substances, which may be associated with an increased cardiometabolic risk [9-11]. As for the association between the serum uric acid levels and the regional abdominal adipose tissue distribution, previous studies showed that the serum uric acid level was related with visceral fat accumulation as measured using computed tomography (CT) or bioelectrical impedance analysis in general populations [12-14].

Diabetes mellitus (DM) is associated with the cardiovascular complications of metabolic syndrome and obesity is more prevalent in people with DM compared in people without DM [15]. Furthermore, the distribution of abdominal adipose tissue was significantly altered in people with type 2 DM; the visceral fat component was greater and the subcutaneous fat component was less in the subjects with type 2 DM than that in the healthy control subjects [16]. The association between the regional distribution of abdominal adipose tissue and the serum uric acid levels in people with type 2 DM is not well established. We hypothesized that the visceral fat component would be more associated with the serum uric acid levels, as compared to that of the subcutaneous fat component, in people with type 2 DM. The aim of our study is to determine the relationships between the regional abdominal adipose tissue distribution and the serum uric acid levels in people with type 2 diabetes mellitus. Examining the influence of the regional abdominal adipose tissue distribution on serum uric acid levels may help to define it as a risk factor for the cardiometabolic complications with type 2 DM.

## Methods

### Study Population

The present study included 699 subjects with type 2 DM and who had undergone abdominal CT for obesity screening at the Bucheon Saint Mary's Hospital between July 2005 and December 2007. Type 2 DM was diagnosed if the patients with a fasting plasma glucose level ≥ 126 mg/dl or a 2-hr post-glucose level after a 75-g oral glucose tolerance test ≥ 200 mg/dl. Patients who were treated by diet alone or in combination with oral hypoglycemic agents or they had fasting serum C-peptide values greater than 1.0 ng/mL when administered insulin were also categorized as type 2 DM. The exclusion criteria were missing values for the body mass index (BMI), the serum uric acid levels or the other clinical variables. Among a total of 953 subjects with type 2 DM and who had undergone abdominal CT for obesity screening, 699 subjects were included in our study. The study protocol was approved by the local ethical committee, and this study was conducted according to the principles of the Declaration of Helsinki.

### Abdominal Adipose Tissue Measurements

Measurements of the cross-sectional abdominal visceral and subcutaneous fat areas by CT (Somatom plus 4, Siemens, Germany) were performed using an established protocol [17]. The subjects were placed supine with their feet first in the scanner and a cross-sectional scan with a 10 mm slice thickness centered at the level of the intervertebral space between the fourth and fifth lumbar vertebrae was obtained with using a radiograph of the skeleton as a reference to establish the position of the scans to the nearest millimeter. The boundaries of the subcutaneous and visceral fat areas were defined by tracing their contours on the scans and the adipose tissue areas were calculated by computing the fat area surfaces with an attenuation range of -190 to -30 Hounsfield units. The visceral fat area was measured within the region by outlining the circumference of the muscle wall surrounding the abdominal cavity. The total fat area was measured in the region by outlining the circumference of the abdominal wall. The subcutaneous fat area was calculated by subtracting the visceral fat area from the total fat area. The measurements were done by one radiologist who was blinded to the subjects' clinical and laboratory data. We tested the intra-observer reproducibility. The visceral fat areas and subcutaneous fat areas of the scans of a subset of 50 randomly selected subjects were repeatedly measured by the radiologist. The intraclass correlation coefficients for the visceral fat areas and subcutaneous fat areas were 0.991 and 0.981, respectively. For the inter-observer reproducibility, previous studies have reported that measurement of the visceral fat areas and subcutaneous fat areas are reproducible between observers [8,18].

### Clinical Information and Laboratory Analysis

The clinical information was assessed from the written and electronic medical records, and this information included the medical history, the current medications and the laboratory data. The collected data included age, gender, the BMI, the systolic and diastolic blood pressures, the duration of diabetes, the serum creatinine, hemoglobin and albumin levels, the total cholesterol and high-density lipoprotein (HDL) cholesterol levels, the triglyceride and serum uric acid levels, the urinary albumin excretion rate, the homeostasis model for insulin resistance (HOMA-IR) score, the lipoprotein (a) and high-sensitivity C-reactive protein (hs-CRP) levels. Fasting venous blood sample were taken for the determination of the serum level of total cholesterol, HDL cholesterol and triglyceride and serum uric acid level was measured on a standard autoanalyzer by the uricase-peroxidase method. Hyperuricemia was defined by cut-off value of > 7 mg/dl for men and > 6 mg/dl for women [19]. The urinary albumin excretion rate was assessed via 24 hour urine collection. The BMI was calculated as weight (kg)/height (m^2^). The blood pressure was measured twice, 5 minutes apart, using a random zero sphygmomanometer with the patient seated after 10 minutes of rest. The estimated GFR (eGFR) was calculated using the Modification of Diet in Renal Disease four-variable equation at the time of CT scanning: eGFR = 186 × serum creatinine^-1.154 ^× age^-0.203 ^× 1.212 (if black) × 0.742 (if female). The HOMA-IR was calculated with the following formula: [Fasting insulin (μIU/mL) × fasting plasma glucose (mmol/L)]/22.5 [20].

### Statistics

The data for the continuous variables with a normal distribution is expressed as means ± SDs and the data for the continuous variables without a normal distribution is expressed as medians with interquartile ranges. The Student's t-test or Mann-Whitney test were used, as appropriate, to determine differences in continuous variables. Categorical variables are presented as percentage. The Pearson's chi-square test was used to determine the differences in categorical variables. Pearson correlation coefficients were used to assess the simple correlation between the regional abdominal adiposity measurements such as the visceral fat area and subcutaneous fat areas and the clinical parameters. Multivariate linear regression analysis was used to determine the association between the abdominal adiposity measurements, including the visceral fat area and the subcutaneous fat area with the serum uric acid levels after adjustment for age, sex, systolic blood pressure, diastolic blood pressure, serum creatinine, hemoglobin, serum albumin, serum HDL cholesterol, serum triglyceride and hemoglobin A1c. Logistic regression analysis was used to estimate odds ratios for hyperuricemia according to the categories of visceral fat areas. *P values *< 0.05 were considered statistically significant. The statistical analyses were performed using SPSS 15.0 software (Chicago, IL, USA).

## Results

### Patients' characteristics

Among a total of 699 subjects with type 2 DM, 389 (55.7%) were females and 310 (44.3%) were males. Table 1 shows the clinical characteristics of the study participants according to gender. The mean age was 55 ± 14 years old. The mean total fat area was 283 ± 125 Cm^2^. The mean visceral fat area was 122 ± 61 Cm^2 ^and the mean subcutaneous fat area was 161 ± 83 Cm^2^. The mean BMI was 25.2 ± 3.9 kg/m^2^. Female had significant higher total fat area and subcutaneous fat area than male (314 ± 121 Cm^2 ^vs 245 ± 118 Cm^2^, p < 0.001 and 191 ± 82 Cm^2 ^vs 122 ± 66 Cm^2^, p < 0.001, respectively). Visceral fat area was not significantly different between female and male (121 ± 58 Cm^2 ^vs 125 ± 66 Cm^2^, p = 0.411). The mean level of serum uric acid was 4.6 ± 1.5 mg/dL. Female had significant lower uric acid levels than male (4.2 ± 1.5 mg/dl vs 5.1 ± 1.5 mg/dl, p < 0.001). Hyperuricemia was note in 95 subjects thus giving an overall prevalence rate of 13.6%. The proportion of hyperuricemia in male subject was higher than that of hyperuricemia in female subject (25% vs 5%, p < 0.001).

**Table 1:** Clinical characteristics of the study participants according to gender

Clinical characteristics	Total (n = 699)	Male (n = 310)	Female (n = 389)	P value
Age, years	55 ± 14	51 ± 13	58 ± 14	< 0.001
BMI, kg/m^2^	25.2 ± 3.9	24.6 ± 3.5	25.5 ± 4.2	0.003
Total fat area, Cm^2^	283 ± 125	245 ± 118	314 ± 121	< 0.001
Visceral fat area, Cm^2^	122 ± 61	125 ± 66	121 ± 58	0.411
Subcutaneous fat area, Cm^2^	161 ± 83	122 ± 66	191 ± 82	< 0.001
Systolic blood pressure, mmHg	131 ± 17	132 ± 17	131 ± 16	0.719
Diastolic blood pressure, mmHg	79 ± 11	79 ± 12	79 ± 10	0.591
Duration of diabetes, months	69 ± 87	50 ± 73	84 ± 94	< 0.001
Serum uric acid, mg/dL	4.6 ± 1.5	5.1 ± 1.5	4.2 ± 1.5	< 0.001
Hyperuricemia, n (%)	95 (14)	77 (25)	18 (5)	< 0.001
Serum creatinine, mg/dL	0.9 ± 0.5	1.0 ± 0.4	0.9 ± 0.3	0.225
Estimated GFR, ml/min/1.73 m^2^	94.4 ± 27.6	95.8 ± 23.1	93.3 ± 30.7	0.243
Hemoglobin, g/dL	13.4 ± 1.9	14.5 ± 2.0	12.6 ± 1.4	< 0.001
Serum albumin, g/dL	4.3 ± 0.5	4.4 ± 0.5	4.3 ± 0.5	< 0.001
Serum total cholesterol, mg/dL	189 ± 45	185 ± 44	192 ± 46	0.048
Serum triglyceride, mg/dL	170 ± 112	181 ± 128	160 ± 95	0.020
Serum HDL, mg/dL	51 ± 15	48 ± 15	53 ± 15	< 0.001
Urinary albumin excretion rate, μg/min	7.1 (4.2-18.7)	7.0 (4.3-19.6)	7.2 (4.1-17.6)	0.785
Hemoglobin A1c	9.0 ± 2.4	9.2 ± 2.5	8.9 ± 2.3	0.100
HOMA-IR	2.5 (1.6-3.9)	2.1 (1.4-3.3)	2.7 (1.8-4.6)	< 0.001
Lipoprotein (a), (IU/L)	170 (50-389)	148 (34-351)	148 (59-402)	0.065
hs-CRP, mg/dL	0.2 (0.1-0.7)	0.2 (0.1-0.9)	0.2 (0.1-0.7)	0.815

Values are expressed as means ± SDs, medians (interquartile range) or n(%).

### Relationships between the serum uric acid levels and the clinical parameters

**Figure 1:**
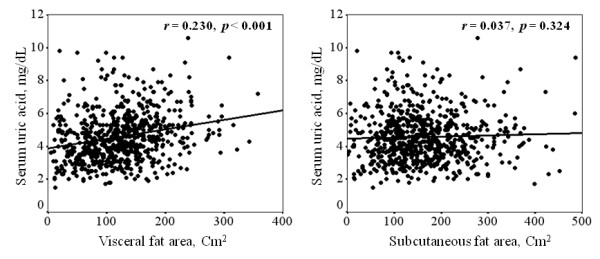
**Pearson's correlation of the visceral fat area and the subcutaneous fat area with the serum uric acid levels in people with type 2 DM**.

We analyzed the relationships between the serum uric acid levels and the clinical parameters (Table 2). The serum uric acid levels were positively correlated with the BMI, the visceral fat area, systolic blood pressure, diastolic blood pressure, serum creatinine, hemoglobin, serum albumin, serum triglyceride, urinary albumin excretion, log HOMA-IR and log hs-CRP. The serum uric acid levels were negatively correlated with eGFR, serum HDL cholesterol and hemoglobin A1c. There was no significant correlation between the serum uric acid levels and the subcutaneous fat area. Figure 1 shows Pearson's correlation of the visceral fat area and the subcutaneous fat area with the serum uric acid levels in people with type 2 DM.

**Table 2:** Correlation coefficients of the relationships between the serum uric acid levels and the clinical parameters

	*r*	*p*
Age	0.015	0.690
BMI	0.182	< 0.001
Visceral fat area	0.230	< 0.001
Subcutaneous fat area	0.037	0.324
Systolic blood pressure	0.123	0.001
Diastolic blood pressure	0.090	0.017
Duration of diabetes	0.032	0.402
Serum creatinine	0.436	< 0.001
Estimated GFR	-0.378	< 0.001
Hemoglobin	0.084	0.027
Serum albumin	0.089	0.019
Serum total cholesterol	-0.018	0.641
Serum triglyceride	0.177	< 0.001
Serum HDL cholesterol	-0.178	< 0.001
Urinary albumin excretion rate	0.110	< 0.001
Hemoglobin A1c	-0.242	< 0.001
Log HOMA-IR	0.090	0.022
Log hs-CRP	0.088	0.026

### Association between the regional abdominal adipose distribution and the serum uric acid levels

Table 3 shows the multivariate linear regression analysis for the association of the regional abdominal adipose tissue distribution with the serum uric acid levels. The visceral fat area was positively associated with serum uric acid levels after adjustment for age, gender, systolic blood pressure, diastolic blood pressure, serum creatinine, hemoglobin, serum albumin, serum HDL cholesterol, serum triglyceride and hemoglobin A1c (β-coefficient = 0.117, *p *< 0.001). The BMI was also positively associated with the serum uric acid levels after adjustment for the clinical parameters (β-coefficient = 0.184, *p *< 0.001). But there was no significant association between the subcutaneous fat area and the serum uric acid levels (β-coefficient = 0.061, *p *= 0.255). We also analyzed the association of visceral fat area tertiles for hyperuricemia in logistic regression model, which included age, gender, systolic blood pressure, diastolic blood pressure, eGFR and serum triglyceride as independent variables (Table 4). The odds ratio (OR) of the third tertile of visceral fat area was 2.33 (95% CI, 1.21-4.50, p = 0.012), which was statistically significant increases for the incidence of hyperuricemia in the group with high visceral fat area.

**Table 3:** Multivariate linear regression analysis for the association of the serum uric acid levels with the regional abdominal adipose tissue distribution

	β-coefficient	P value
Visceral fat area, Cm	0.117	< 0.001
Subcutaneous fat area, Cm	0.061	0.255
BMI, kg/m^2^	0.184	< 0.001

Adjusted for age, sex, systolic blood pressure, diastolic blood pressure, serum creatinine, hemoglobin, serum albumin, serum high-density lipoprotein, serum triglyceride and hemoglobin A1c

**Table 4:** Multivariate logistic regression analyses to determine the predictor of hyperuricemia

Clinical variables	Odds ratio	95% CI	P value
Age	1.0	0.97-1.02	0.638
Gender (male)	13.58	6.56-28.18	< 0.001
Systolic blood pressure	1.01	0.99-1.03	0.377
Diastolic blood pressure	0.99	0.96-1.03	0.632
Estimated GFR < 6o ml/min/1.73 m^2^	16.75	7.06-39.77	< 0.001
Serum triglyceride	1.00	1.00-1.00	0.153
Visceral fat area tertiles			
I (< 93 Cm^2^)	1		
II (93 - 143 Cm^2^)	1.50	0.76-2.97	0.245
III (> 143 Cm^2^)	2.33	1.21-4.50	0.012

## Discussion

In the present study, we demonstrated that the visceral fat area were positively associated with the serum uric acid levels and predictive for the hyperuricemia in subjects with type 2 DM, but the subcutaneous fat area was not significantly associated with the serum uric acid levels. In our knowledge, this is a first report for the association between the regional abdominal adipose tissue distribution and the serum uric acid levels in subjects with type 2 DM, in which the distribution of the abdominal adipose tissue was significantly altered as compared to that of the subjects with normal glucose tolerance. The amount of visceral fat area was greater and the amount of subcutaneous fat area was less in people with type 2 DM than that in the people with normal glucose tolerance [16].

Some previous studies that focused on general populations reported that the serum uric acid levels were positively correlated with both the visceral fat area and the subcutaneous fat area; particularly, the serum uric acid levels were more closely correlated with the visceral fat [12-14]. Matsuura et al. reported that the serum uric acid levels were higher in both the subcutaneous obesity group and the visceral obesity group of male obese subjects than that in the non-obese control group [12]. They also showed that urinary uric acid excretion was higher in the visceral obesity group than that in the subcutaneous obesity group, which suggested that visceral fat accumulation may be associated with overproduction of uric acid. Hikita et al reported that the serum uric acid levels were related with both the visceral fat area and the subcutaneous fat area in 508 Japanese man industrial workers, which support the results of previous study [14]. In our study, only the visceral fat area was associated with the serum uric acid levels and subcutaneous fat area was not associated with the serum uric acid levels, which is some different from the previous studies. This discrepancy may be due to the differences of the study designs or the populations of the studies. In our study, only subjects with type 2 DM were recruited while the previous study was a community-based study. The amount of visceral fat area was greater and the amount of subcutaneous fat area was less in people with type 2 DM than that in the people with normal glucose tolerance [16]. Furthermore, visceral fat area, but not subcutaneous fat area, is associated with a decrease in peripheral insulin sensitivity in type 2 DM [21], which may decrease the urinary excretion of uric acid and this may causes hyperuricemia especially in the subjects with type 2 DM [22,23].

Some studies have suggested the mechanisms that link visceral fat accumulation and an elevated serum uric acid level. The visceral fat component has been regarded as the more pathologic adipose tissue compartment as compared with the subcutaneous fat component, and the visceral fat component more strongly associated with the cardiometabolic risk, as compared to that of subcutaneous adiposity [8,9]. The visceral fat component is metabolically active and it regulates numerous adipocytokines such as leptin and adiponectin, which have been associated with insulin resistance [24,25]. Insulin resistance or hyperinsulinemia increases the reabsorption of sodium and uric acid on the renal tubules, thereby decreasing the urinary excretion of uric acid and this causes hyperuricemia [22,23]. In our study, the insulin resistance (log HOMA-IR) was significantly correlated with the serum uric acid levels, which supports the results of previous studies [22,23].

Another mechanism that visceral fat accumulation is associated with the overproduction of uric acid has been suggested by several investigators. Increased visceral fat accumulation provides excessive free fatty acid in the portal vein, which accelerates the overproduction of very low-density lipoprotein and this causes hypertriglyceridemia. This also accelerates the de novo purine synthesis by NADPH produced in the pentose phosphate pathway which increases the uric acid production [26,27]. In our study, the visceral fat area was more closely correlated with the serum triglyceride levels (*r *= 0.244, *p *< 0.001*) *than the subcutaneous fat area (*r *= 0.094, *p *= 0.012) (data not shown). The serum triglyceride levels were positively associated with the serum uric acid levels after adjustment for age, sex, systolic blood pressure, diastolic blood pressure, BMI, serum creatinine, hemoglobin, serum albumin, serum HDL, serum triglyceride, urinary albumin excretion and hemoglobin A1c (β-coefficient = 0.109, *p *= 0.003) (data not shown), and this all supports the relationships between uric acid production and triglyceride synthesis.

Previous studies demonstrated that the reduced GFR was correlated with hyperuricemia [28]. In our study, eGFR was negatively related with serum uric acid levels and the association was stronger between eGFR and serum uric acid levels (r = -0.378, p < 0.001) than the association between visceral fat area and serum uric acid levels (r = 0.230, p < 0.001) (Table 2). To determine whether the visceral fat area was associated with serum uric acid levels independent of the reduced renal function, we analyzed the association between visceral fat area and serum uric acid levels in patients with normal renal function (eGFR > 6o ml/min/1.73 m^2^). The number of patients with eGFR > 6o ml/min/1.73 m^2 ^was 629 (90%) in our study. In patients with eGFR > 6o ml/min/1.73 m^2^, the association between visceral fat area and serum uric acid levels was stronger (r = 0.290, p < 0.001) than the association between eGFR and serum uric acid levels (r = - 0.269, p < 0.001) (data not shown). Furthermore, in the multivariate logistic regression analyses, visceral fat area was independently associated with serum uric acid levels (Table 4). These findings suggest that the visceral fat area was associated with serum uric acid levels independent of the reduced renal function.

In our study, hyperuricemia is more prevalent in males than in females, which was consistent with previous studies [19]. This finding may be due that estrogen promotes more efficient renal clearance of uric acid [29]. It would be interesting to analyze the associations between hyperuricemia and the regional abdominal adipose tissue distribution separately in males and females. However, in our study, the sample size of subjects with hyperuricemia in females (n = 18) was relatively small to generate the significant results by statistical analysis. Therefore, we analyzed the association of visceral fat area tertiles for hyperuricemia in both genders in logistic regression model including gender as independent variables (Table 4). The incidence of hyperuricemia was statistically significant increases in the group with high visceral fat area after adjustment of clinical variables including gender. A larger study may elucidate the associations separately in males and females.

Our study has several limitations. First, our study was the cross-sectional study; therefore, it is difficult to infer causality between visceral fat accumulation and the serum uric acid levels. Second, this study was a single center study; thus, it is uncertain whether our results are generalizable to other ethnic groups with type 2 DM. Third, we did not examined urinary uric acid levels in our study. Therefore, we could not analyze the association between uric acid metabolism or the type of hyperuricemia and the regional distribution of abdominal adipose tissue. Fourth, smoking habit or adipokines such as leptin may influence the distribution of regional abdominal adiposity and insulin resistance [30,31]. Unfortunately, the clinical information in our study did not include the smoking history or adipokines. It would be interesting to analyze the associations between smoking habit or adipokines levels and abdominal adiposity and serum uric acid levels.

Despite the above limitations, the present study is the first to investigate the differential association between the abdominal fat compartment and the serum uric acid levels in people with type 2 DM and who have a different regional abdominal adipose tissue distribution compared to that of people with normal glucose tolerance.

## Conclusions

The visceral fat area was positively associated with the serum uric acid levels, but the subcutaneous fat area was not in people with type 2 DM. Our data suggests that the visceral adiposity may be more metabolically active and it may be associated with an increased risk of hyperuricemia, as compared to that of subcutaneous adiposity in people with type 2 DM. Furthermore, pervious study reported that the changes of serum uric acid were correlated with the changes of visceral fat thickness but not with the changes of subcutaneous fat thickness 1 year after bariatric restrictive surgery in morbidly obese subjects [32]. Therefore, it may be postulated that the efforts to decrease the visceral fat accumulation may be helpful to prevent hyperuricemia and reduce the risk of cardiovascular disease in people with type 2 DM [32,33].

## Competing interests

The authors declare that they have no competing interests.

## Authors' contributions

THK, SSL, JHY, SRK, SJY, HCS, YSK, EJC contributes in execution, analysis, manuscript drafting and critical discussion. YKK contributes in study design, execution, analysis, manuscript drafting and critical discussion. All authors read and approved the final manuscript.

## Authors' details

Department of Internal Medicine, College of Medicine, The Catholic University of Korea, Seoul, Korea

## References

[B1] Fang J Alderman MH Serum uric acid and cardiovascular mortality the NHANES I epidemiologic follow-up study, 1971-1992. National Health and Nutrition Examination Survey JAMA 2000 283 2404 2410 10.1001/jama.283.18.2404 10815083

[B2] Lehto S Niskanen L Rönnemaa T Laakso M Serum uric acid is a strong predictor of stroke in patients with non-insulin-dependent diabetes mellitus Stroke 1998 29 635 639 10.1161/01.STR.29.3.635 9506605

[B3] Vuorinen-Markkola H Yki-Järvinen H Hyperuricemia and insulin resistance J Clin Endocrinol Metab 1994 78 25 29 10.1210/jc.78.1.25 8288709

[B4] Cigolini M Targher G Tonoli M Manara F Muggeo M De Sandre G Hyperuricaemia: relationships to body fat distribution and other components of the insulin resistance syndrome in 38-year-old healthy men and women Int J Obes Relat Metab Disord 1995 19 92 96 7735346

[B5] Onat A Uyarel H Hergenç G Karabulut A Albayrak S Sari I Yazici M Keleş I Serum uric acid is a determinant of metabolic syndrome in a population-based study Am J Hypertens 2006 19 1055 1062 10.1016/j.amjhyper.2006.02.014 17027827

[B6] Costa A Igualá I Bedini J Quintó L Conget I Uric acid concentration in subjects at risk of type 2 diabetes mellitus: relationship to components of the metabolic syndrome Metabolism 2002 51 372 375 10.1053/meta.2002.30523 11887176

[B7] Ogbera AO Azenabor AO Hyperuricaemia and the metabolic syndrome in type 2 DM Diabetol Metab Syndr 2010 2 24 10.1186/1758-5996-2-24 20406485 PMC2864200

[B8] Fox CS Massaro JM Hoffmann U Pou KM Maurovich-Horvat P Liu CY Vasan RS Murabito JM Meigs JB Cupples LA D'Agostino RB Sr O'Donnell CJ Abdominal visceral and subcutaneous adipose tissue compartments: association with metabolic risk factors in the Framingham Heart Study Circulation 2007 116 39 48 10.1161/CIRCULATIONAHA.106.675355 17576866

[B9] Hayashi T Boyko EJ Leonetti DL McNeely MJ Newell-Morris L Kahn SE Fujimoto WY Visceral adiposity is an independent predictor of incident hypertension in Japanese Americans Ann Intern Med 2004 140 992 1000 15197016 10.7326/0003-4819-140-12-200406150-00008

[B10] Mertens I Van Gaal LF Visceral fat as a determinant of fibrinolysis and hemostasis Semin Vasc Med 2005 5 48 55 10.1055/s-2005-871741 15968580

[B11] Miyazawa-Hoshimoto S Takahashi K Bujo H Hashimoto N Saito Y Elevated serum vascular endothelial growth factor is associated with visceral fat accumulation in human obese subjects Diabetologia 2003 46 1483 1488 10.1007/s00125-003-1221-6 14534780

[B12] Matsuura F Yamashita S Nakamura T Nishida M Nozaki S Funahashi T Matsuzawa Y Effect of visceral fat accumulation on uric acid metabolism in male obese subjects: visceral fat obesity is linked more closely to overproduction of uric acid than subcutaneous fat obesity Metabolism 1998 47 929 933 10.1016/S0026-0495(98)90346-8 9711987

[B13] Tamba S Nishizawa H Funahashi T Okauchi Y Ogawa T Noguchi M Fujita K Ryo M Kihara S Iwahashi H Yamagata K Nakamura T Shimomura I Matsuzawa Y Relationship between the serum uric acid level, visceral fat accumulation and serum adiponectin concentration in Japanese men Intern Med 2008 47 1175 1180 10.2169/internalmedicine.47.0603 18591837

[B14] Hikita M Ohno I Mori Y Ichida K Yokose T Hosoya T Relationship between hyperuricemia and body fat distribution Intern Med 2007 46 1353 1358 10.2169/internalmedicine.46.0045 17827832

[B15] Iseki K Predictors of diabetic end-stage renal disease in Japan Nephrology (Carlton) 2005 10 Suppl S2 S6 16174282 10.1111/j.1440-1797.2005.00447.x

[B16] Gallagher D Kelley DE Yim JE Spence N Albu J Boxt L Pi-Sunyer FX Heshka S MRI Ancillary Study Group of the Look AHEAD Research Group Adipose tissue distribution is different in type 2 diabetes Am J Clin Nutr 2009 89 807 814 10.3945/ajcn.2008.26955 19158213 PMC2714397

[B17] Ferland M Després JP Tremblay A Pinault S Nadeau A Moorjani S Lupien PJ Thériault G Bouchard C Assessment of adipose tissue distribution by computed axial tomography in obese women: association with body density and anthropometric measurements Br J Nutr 1989 61 139 148 10.1079/BJN19890104 2706220

[B18] Tirkes AT Gottlieb RH Voci SL Waldman DL Masetta J Conover DL Risk of significant coronary artery disease as determined by CT measurement of the distribution of abdominal adipose tissue J Comput Assist Tomogr 2002 26 210 215 10.1097/00004728-200203000-00009 11884776

[B19] Sui X Church TS Meriwether RA Lobelo F Blair SN Uric acid and the development of metabolic syndrome in women and men Metabolism 2008 57 845 852 10.1016/j.metabol.2008.01.030 18502269 PMC2486830

[B20] Haffner SM Miettinen H Stern MP The homeostasis model in the San Antonio Heart Study Diabetes Care 1997 20 1087 1092 10.2337/diacare.20.7.1087 9203442

[B21] Gastaldelli A Miyazaki Y Pettiti M Matsuda M Mahankali S Santini E DeFronzo RA Ferrannini E Metabolic effects of visceral fat accumulation in type 2 diabetes J Clin Endocrinol Metab 2002 87 5098 5103 10.1210/jc.2002-020696 12414878

[B22] Quiñones Galvan A Natali A Baldi S Frascerra S Sanna G Ciociaro D Ferrannini E Effect of insulin on uric acid excretion in humans Am J Physiol 1995 268 E1 E5 7840165 10.1152/ajpendo.1995.268.1.E1

[B23] Ter Maaten JC Voorburg A Heine RJ Ter Wee PM Donker AJ Gans RO Renal handling of urate and sodium during acute physiological hyperinsulinaemia in healthy subjects Clin Sci (Lond) 1997 92 51 58 9038591 10.1042/cs0920051

[B24] Wajchenberg BL Subcutaneous and visceral adipose tissue: their relation to the metabolic syndrome Endocr Rev 2000 21 697 738 10.1210/er.21.6.697 11133069

[B25] Goodpaster BH Krishnaswami S Resnick H Kelley DE Haggerty C Harris TB Schwartz AV Kritchevsky S Newman AB Association between regional adipose tissue distribution and both type 2 diabetes and impaired glucose tolerance in elderly men and women Diabetes Care 2003 26 372 379 10.2337/diacare.26.2.372 12547865

[B26] Fabregat I Revilla E Machado A Short-term control of the pentose phosphate cycle by insulin could be modulated by the NADPH/NADP ratio in rat adipocytes and hepatocytes Biochem Biophys Res Commun 1987 146 920 925 10.1016/0006-291X(87)90618-8 3304289

[B27] Matsubara K Matsuzawa Y Jiao S Takama T Kubo M Tarui S Relationship between hypertriglyceridemia and uric acid production in primary gout Metabolism 1989 38 698 701 10.1016/0026-0495(89)90110-8 2739579

[B28] Neri L Rocca Rey LA Lentine KL Hinyard LJ Pinsky B Xiao H Dukes J Schnitzler MA Joint association of hyperuricemia and reduced GFR on cardiovascular morbidity: a historical cohort study based on laboratory and claims data from a national insurance provider Am J Kidney Dis 2011 58 398 408 10.1053/j.ajkd.2011.04.025 21783292

[B29] Hak AE Choi HK Menopause, postmenopausal hormone use and serum uric acid levels in US women--the Third National Health and Nutrition Examination Survey Arthritis Res Ther 2008 10 R116 10.1186/ar2519 18822120 PMC2592803

[B30] Matsushita Y Nakagawa T Yamamoto S Takahashi Y Noda M Mizoue T Associations of smoking cessation with visceral fat area and prevalence of metabolic syndrome in men: the Hitachi health study Obesity (Silver Spring) 2011 19 647 651 10.1038/oby.2010.237 20966912

[B31] Lin JD Chiou WK Chang HY Liu FH Weng HF Serum uric acid and leptin levels in metabolic syndrome: a quandary over the role of uric acid Metabolism 2007 56 751 756 10.1016/j.metabol.2007.01.006 17512306

[B32] Pontiroli AE Frigè F Paganelli M Folli F In morbid obesity, metabolic abnormalities and adhesion molecules correlate with visceral fat, not with subcutaneous fat: effect of weight loss through surgery Obes Surg 2009 19 745 750 10.1007/s11695-008-9626-4 18629594

[B33] Alexandrides TK Skroubis G Kalfarentzos F Resolution of diabetes mellitus and metabolic syndrome following Roux-en-Y gastric bypass and a variant of biliopancreatic diversion in patients with morbid obesity Obes Surg 2007 17 176 184 10.1007/s11695-007-9044-z 17476868

